# Voxel-Based MRI Intensitometry Reveals Extent of Cerebral White Matter Pathology in Amyotrophic Lateral Sclerosis

**DOI:** 10.1371/journal.pone.0104894

**Published:** 2014-08-18

**Authors:** Viktor Hartung, Tino Prell, Christian Gaser, Martin R. Turner, Florian Tietz, Benjamin Ilse, Martin Bokemeyer, Otto W. Witte, Julian Grosskreutz

**Affiliations:** 1 Hans-Berger Department of Neurology, University Hospital Jena, Jena, Germany; 2 Department of Psychiatry and Psychotherapy, University Hospital Jena, Jena, Germany; 3 Department of Radiology, Section Neuroradiology, University Hospital Jena, Jena, Germany; 4 Nuffield Department of Clinical Neurosciences, Oxford University, Oxford, United Kingdom; 5 Center for Sepsis Control and Care (CSCC), University Hospital Jena, Jena, Germany; 6 Department of Palliative Medicine, University Medical Centre, Goettingen, Germany; Banner Alzheimer's Institute, United States of America

## Abstract

Amyotrophic lateral sclerosis (ALS) is characterized by progressive loss of upper and lower motor neurons. Advanced MRI techniques such as diffusion tensor imaging have shown great potential in capturing a common white matter pathology. However the sensitivity is variable and diffusion tensor imaging is not yet applicable to the routine clinical environment. Voxel-based morphometry (VBM) has revealed grey matter changes in ALS, but the bias-reducing algorithms inherent to traditional VBM are not optimized for the assessment of the white matter changes. We have developed a novel approach to white matter analysis, namely voxel-based intensitometry (VBI). High resolution T1-weighted MRI was acquired at 1.5 Tesla in 30 ALS patients and 37 age-matched healthy controls. VBI analysis at the group level revealed widespread white matter intensity increases in the corticospinal tracts, corpus callosum, sub-central, frontal and occipital white matter tracts and cerebellum. VBI results correlated with disease severity (ALSFRS-R) and patterns of cerebral involvement differed between bulbar- and limb-onset. VBI would be easily translatable to the routine clinical environment, and once optimized for individual analysis offers significant biomarker potential in ALS.

## Introduction

Amyotrophic lateral sclerosis (ALS) is a fatal neurodegenerative disease characterized by progressive loss of upper motor neurons (UMN) and lower motor neurons (LMN). It is now regarded as a multi-system disease, showing consistent extra-motor involvement and has genetic, clinical and pathological overlap with frontotemporal dementia (FTD) [1], [2]. The firm clinical diagnosis of ALS depends upon the demonstration of UMN as well as LMN involvement. Signs of LMN involvement such as atrophic paresis may obscure UMN symptoms and contribute to the delay in diagnosis, initiation of riluzole therapy and potentially enrolment into therapeutic trials.

Biomarkers are urgently sought in ALS [3]. Markers sensitive to UMN involvement would have particular value as UMN signs are not always be clinically obvious [4]. Routine clinical MRI sequences may reveal corticospinal tract (CST) hyperintensity, but this is not sensitive or specific for ALS [5]. Diffusion tensor imaging (DTI) has defined a core white matter pathology, involving the CST and corpus callosum in ALS [6], [7], it is not yet a routine clinical sequence and will be challenging to harmonize across international centres.

Voxel-based morphometry (VBM) is an automated MRI analysis technique typically used to demonstrate regional differences in grey matter. In ALS it has revealed widespread changes, clearly extending beyond the motor areas with involvement of frontal, temporal parietal and limbic cortices [8]–[14]. Moreover the focal grey matter atrophy within the motor homunculus appears to correspond with regional functional disability [15]. To date, however the sensitivity of VBM to detect ALS related pathology has been limited [16].

VBM uses prior knowledge of tissue composition and distribution to assign a probability to every image voxel of belonging to gray matter, white matter or cerebrospinal fluid [17], [18]. Standard VBM is not particularly suited to assess deep white matter alterations, because it cannot handle subtle intensity changes within large areas of uniform tissue. If image preprocessing steps like segmentation and spatial normalization work well, voxels within white matter areas are considered uniform, and are thus given equal values that remove any contrast that might be generated from CST changes in ALS. We have developed a contrast enhancement technique, named voxel-based intensitometry (VBI) that is sensitive to deep white matter tissue changes in T1-weighted MRI. We applied VBI to a large group of ALS patients in comparison to age-matched healthy controls, to demonstrate its ability to detect widespread cerebral white matter tract involvement.

## Methods

### Participants

ALS patients (n = 30) from the Hans-Berger Department of Neurology (University hospital Jena) were enrolled into the study. Diagnosis of ALS was made according to the revised El-Escorial criteria by experienced ALS neurologists (JG, TP). All patients were established on riluzole and none were taking psychoactive drugs. Disability was assessed using the revised ALS Functional Rating Score (ALSFRS-R). Those with manifest cognitive deficits were excluded using Mini Mental State Examination (MMSE) and Frontal Assessment Battery (FAB). The 37 age-matched healthy controls had no history of neurological disease, normal neurological examination, and MRI T1 and T2-weighted images did not reveal any pathological findings. Clinical characteristics are summarized in table 1. All participants gave written informed consent to participate in the study, which was approved by the local research ethics committee (Ethik-Kommission Universitätsklinikum Jena, EK 3619-11/12).

**Table 1:** Clinical characteristics of participants.

	Age (y)	sex f:m	Disease duration (month)	Handedness (left: right)	ALSFRS-R	ALSFRS-R subscores			
						bulbar	cervical	lumbar	thoracic
**All patients (n = 30)**									
Mean	62.7	13∶17	23.5	02∶28	37.1	10	8	7.9	11.3
S.D.	±11.2		±18.1		±6.1	±2.4	±3.1	±3	±1.3
**Bulbar-onset (n = 13)**									
Mean	62	8∶5	19.4	01∶12	38.2	8.2	9	10	11
S.D.	±12.4		±18.2		±6.7	±2.5	±2.9	±2.2	±0.9
n	13								
**Limb-onset (n = 17)**									
Mean	63.2	5∶12	25.6	1∶16	36.3	11.3	7.1	6.3	11.6
S.D.	±10.7		±16.6		±5.7	±1	±3	±2.6	±1.5
n	17								
**bulbar vs. limb**									
P value (U test)	n.s.	n.s.	n.s.	n.s.	n.s.	0.001	0.1	0.001	0.01
**low disability**									
Mean	57.9	8∶8	18.3	0∶16	41.8	10.6	10	9.6	11.6
S.D.	±12.1		±17.1		±2.8	±1.9	±1.7	±2.3	±0.8
n	16								
**high disability**									
Mean	68.2	5∶9	28.1	2∶12	31.9	9.3	5.6	6	11
S.D.	±7.1		±16.6		±4.2	±2.7	±2.5	±2.6	±2.7
n	14								
**low vs. high disability**									
P value (U test)	0.01	n.s.	n.s.	n.s	0.001	0.001	0.1	0.001	0.01
**Controls (n = 37)**									
Mean	60.8	18∶19							
S.D.	12.1								

### Data acquisition and preprocessing

High resolution T1-weighted FLASH 3D scans were obtained on a 1.5 Tesla Siemens Sonata scanner acquiring 192 sagittal slices (field of view of 224×256×192 pixels, TR = 15 ms, TE = 5 ms, Flip Angle = 30°) and using a standard 4-channel headcoil. All participants were comfortably placed and their heads secured using cushions during scanning to minimize motion artefact. Voxel-wise analysis and statistics was performed using Statistical Parametric Mapping (SPM8, Wellcome Trust Centre for Neuroimaging, UCL, London, UK), within a Matlab framework (The MathWorks Inc., Natick MA, USA) in its R2009b iteration. Image calculations like *mean* or *average* were done using basic operations that are provided within SPM8 and Matlab.

DICOMs were converted into the Nifti format by using Dcm2Nii (MRIcroN). For preprocessing we used the VBM8-toolbox for SPM8 and Matlab, which is publicly available under the GNU General Public License (http://dbm.neuro.uni-jena.de/vbm/download/), because this toolbox comprises improvements over the standard SPM8 algorithms (e.g. segmentation without tissue priors, integration of Dartel normalization). Tissue probability maps for grey matter, white matter and corticospinal fluid and deformation fields were calculated by using the default settings of the toolbox (VBM8: Estimate & Write, default, Deformation Fields: forward).

In the next step all spatially normalized white matter segments were averaged and thresholded at the ninth percentile to obtain a common space in which inference is searched for. This white mask was later used as explicit mask in the statistical analysis (SPM > Stats > Factorial design specification > explicit mask). The deformation fields, obtained from the VBM preprocessing were used to normalize the original T1-weighted scans into MNI space (SPM > Tools > VBM8 > Tools > Apply deformations).

### Intensity normalization

**Figure 1:**
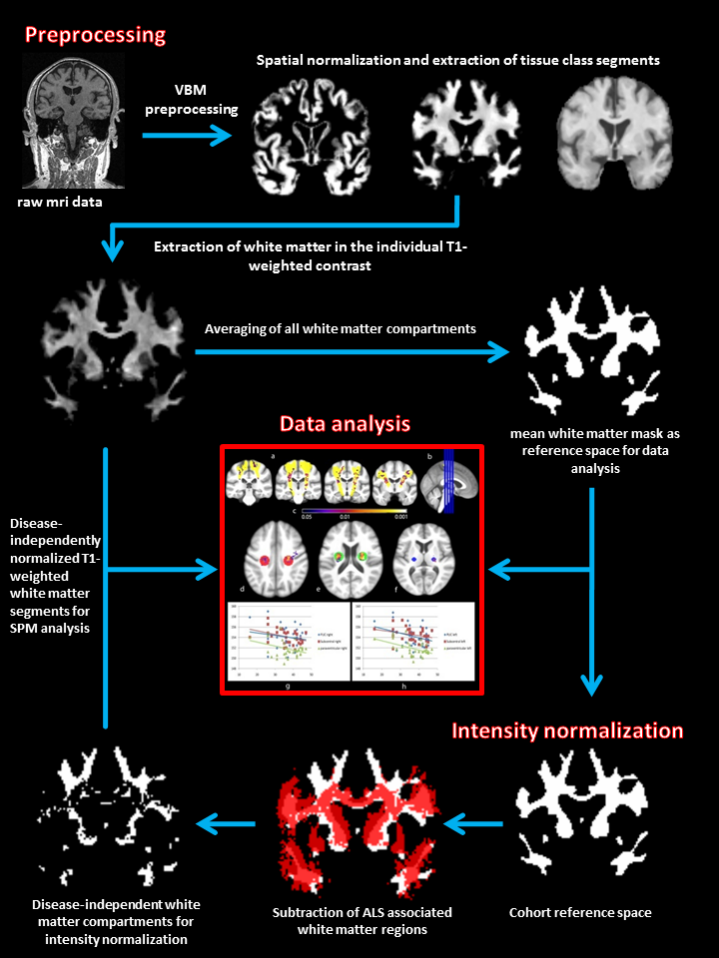
Processing algorithm. Flowchart illustrates the processing algorithm used for the analyses. VBM-based spatial preprocessing is combined with disease-independent intensity normalization to allow for maximal standardization and direct comparability of T1-contrasted MRI images.

Given the prior knowledge from neuropathology and DTI studies (see introduction) we used the JHU white-matter MRI atlas to create a mask of suspected ALS-related white-matter partitions: the CST, inferior frontooccipital fasciculus, superior and inferior longitudinal fasciculus, anterior, superior and posterior corona radiata, anterior and posterior limb of the internal capsule, genu, corpus and splenium of the corpus callosum, cerebral peduncle, and cingulate gyrus. These regions were then subtracted from the study-specific white matter mask. Thus we extracted the overall white matter brightness level of regions, which are not affected by ALS pathology. Then the T1 white matter intensity of each scan was brightness standardized by multiplying the resulting factor to an arbitrary mean value. Therefore the resulting images were disease-independently normalized in their intensity levels, making global covariate correction in the following ANCOVAs unnecessary. A flowchart of the preprocessing steps is given in **figure 1**.

### Statistical analysis

All statistical analyses were done using the SPM approach. Threshold-free cluster enhancement (TFCE) with family-wise error (FWE) correction was used to control for multiple comparisons [19]. TFCE allows increases sensitivity and specificity of results and reduces their arbitrariness as individual definitions of cluster extent values (minimal cluster size of response for recognition as real effect) and therefore thresholds for minimal significance levels per voxel are not necessary (SPM>Stats>Factorial design specification>Model estimation>TFCE).

In order to explore the correlation between white matter intensities and the functional state of ALS patients, regression analysis was conducted between the ALSFRS-R scores and CST-related ROIs in the posterior limb of the internal capsule (PLIC), periventricular and subcentral regions (linear regression, ANOVA). The mean intensity in these regions was then plotted against the ALSFRS-R for each subject. A *post hoc* analysis comparing approximately equally sized groups either side of an ALSFRS-R score of 36 (‘low’ versus ‘high’ disability) was undertaken: group 1 ALSFRS-R range 37–47 (n = 16); group 2 ALSFRS-R range 26–36 (n = 14).

In order to explore differences in VBI patterns between ALS patients with bulbar- versus limb-onset, these groups were separately compared to controls. The groups did not significantly differ in age, sex, disease duration or ALSFRS-R (table 1).

### Effect size

For coarse estimation of MRI intensity response to disease we first computed the mean intensity range inside the common white matter space in all participants as reference to evaluate the relative change in intensity (range between brightest and darkest intensity as total spread). All intensity changes are referenced to the mean value of the intensity normalization mask (white matter of non ALS-related regions) and the total spread (relative change to mean, given in percent). The representative regions inside the CST were analyzed. Next, we defined ROIs outside these regions as control. These were in the frontoparietal white matter (sphere, radius of 5 mm; coordinates (X, Y, Z) in MNI space, left: 80, 85, 76; right: 44, 85, 76) and in the parietooccipital white matter (left: 85, 52, 70; right: 38, 52, 70).

## Results

### Group comparison

**Figure 2:**
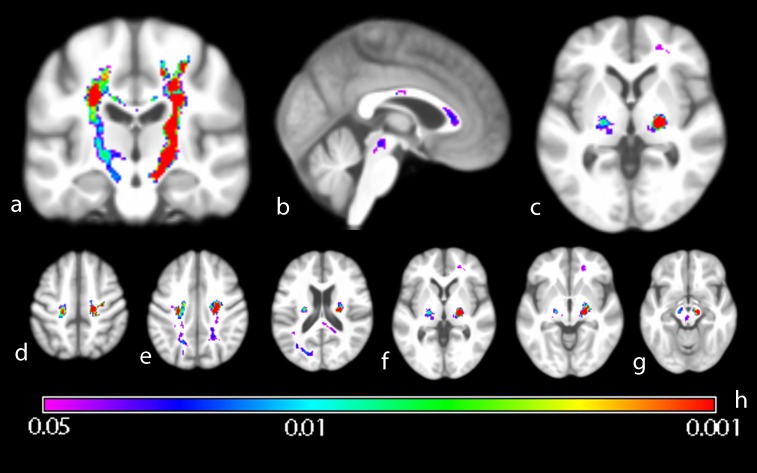
Group comparison of ALS patients versus healthy controls. Wholebrain group-mean of VBI images projected on generic brain. Coronal (a), sagittal (b) und axial (c) sections illustrate significantly higher intensity in the CST, corpus callosum and posterior limb of the internal capsule (PLIC). Significant VBI increases were see descending from the motor-cortical level (d) into the cerebral peduncle, rendering areas like the radiate corona (e), PLIC (f) to the midbrain nuclei (g). Inference was done using Threshold Free Cluster Enhancement (10000 permutations) und Family-wise error rate correction for multiple comparisons. Color spectrum gives p-value indication (h).

Widespread increases in white matter intensity were seen in the ALS group, prominent in the CSTs bilaterally and corpus callosum. To various degrees other structures were also involved. These were the anterior, superior and posterior radiate corona, major and minor fornix, the temporal part of the superior longitudinal fasciculus, anterior thalamic radiation and the inferior longitudinal and frontooccipital fasciculus (**figure 2**).

The mean extent of significant intensity increase ranges from 4.1% in the left subcentral region to 8.7% in the PLIC (table 2). Reference regions outside ALS-related white matter compartments did not significantly differ between patients and controls.

**Table 2:** Intensity response in key CST areas and control regions.

Absolute difference to controls (%)	PLIC left	PLIC right	Para-ventricular left	Para-ventricular right	Subcentral left	Subcentral right	frontal left	frontal rigth	occipital left	occipital right
low disability	7.2***	4.8*	2.2 n.s.	0.8 n.s.	2.7*	3.2*	−2.0 n.s.	−2.1 n.s.	1.2 n.s.	1.6 n.s.
high disability	8.7***	5.5 n.s.	6.2*	4.6 n.s.	5.7**	6.4**	0.0 n.s.	−1.6 n.s.	2.8 n.s.	−1.1 n.s.
all patients	8.7***	5.1**	4.1**	2.6 n.s.	4.1***	4.7***	−1.1 n.s.	−1.8 n.s.	2.4 n.s.	0.3 n.s.

Mean intensity inside ROIs along the CST and in ALS independent white matter regions are shown. The absolute difference of the mean in patients versus controls is referenced to the total white matter intensity spread to allow comparability to other study settings. Significances are given as * (p<0.05), ** (p<0.01), ***(P<0.001) or as not significant (n.s.). The highest response was found in the left PLIC (8.7% higher intensity in patients than in controls). The non ALS disease related white matter regions did not significantly differ between patients and controls.

### Correlation with disease severity

**Figure 3:**
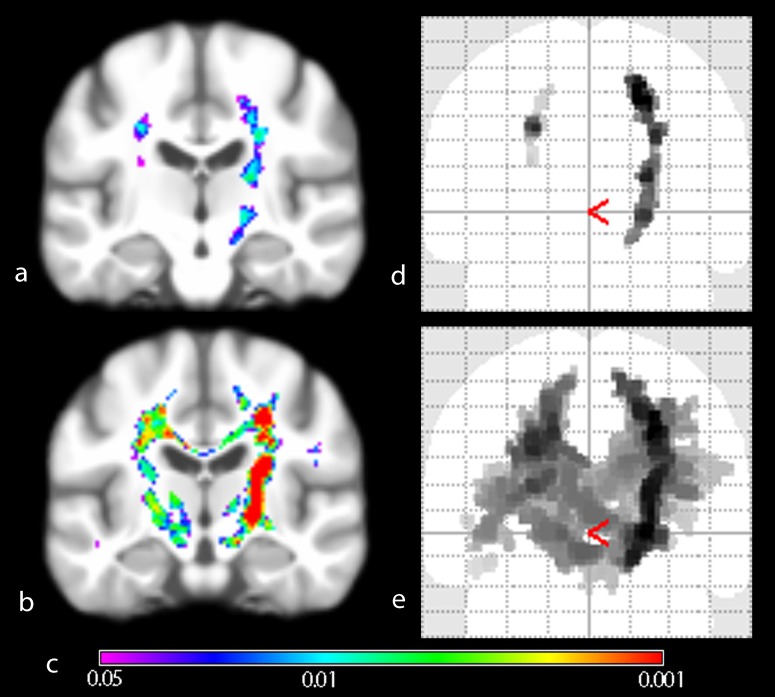
Group comparison of ALSFRS-R stratified patient subgroups versus healthy controls. Wholebrain group-mean of VBI images projected on generic brain (a, b) and Maximum Intensity Projection of significantly different regions (d, e). The extent of MRI intensity change in ‘low’ disability group (a, d) is significantly less widespread than in the ‘high’ disability group (b, e). Inference was done using Threshold Free Cluster Enhancement (10000 permutations) und Family-wise error rate correction for multiple comparisons. Color spectrum giving p-value indication (c).

**Figure 4:**
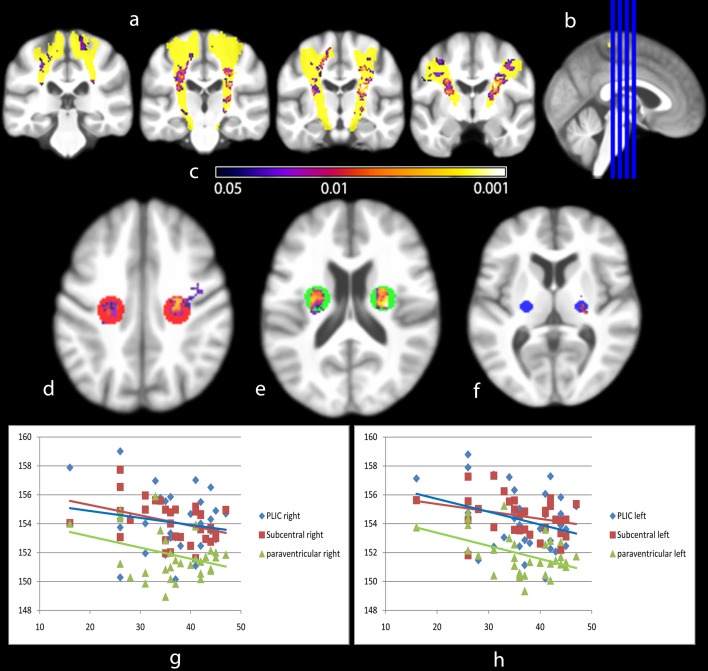
Regression analysis of VBI-processed T1 intensities of all ALS patients and their ALSFRS-R. Clusters of significant correlation between VBI intensity and ALSFRS-R scores are given in coronal slices (a, level at b). Projection on VBI group mean generic brain, CST highlighted according to JHU DTI atlas (yellow). Mean intensity inside ROIs in the PLIC (f, 5 mm sphere), paraventricular (e, 10 mm sphere) and subcentral white matter (d, 10 mm sphere) reveals overall decrease in intensity (mean VBI intensity versus ALSFRS-R; g, right; h, left). This illustrates a disability-related MRI intensity change in ‘core’ regions of ALS-related white matter disturbances. Inference was done using Threshold Free Cluster Enhancement (10000 permutations). Color spectrum giving p-value indication (c).

The ALSFRS-R ‘low’ and ‘high’ disability group comparisons showed expanding intensity gain with increasing disability (**figure 3**). Using regression analysis, mean intensity in key CST areas correlated significantly with the ALSFRS-R (R = −0.73, p = 0.019, degrees of freedom 29, **figure 4**).

### ALS subtype comparison

**Figure 5:**
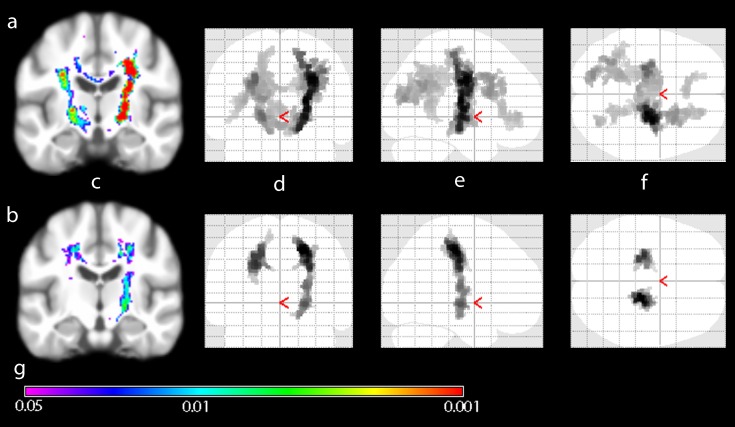
Group comparison of bulbar and limb phenotype patient subgroups versus healthy controls. Wholebrain group-mean of VBI images projected on generic brain (c) and Maximum Intensity Projection (MIP) of significantly different regions (d, e, f). The patterns of MRI intensity change in bulbar (a) and limb (b) subgroups significantly differ despite no significant differences in age, sex or ALSFRS-R score between groups. Coronal (d), sagittal (e) and axial (f) MIPs illustrate apparently more widespread involvement of cerebral white matter in bulbar-onset ALS. Inference was done using Threshold Free Cluster Enhancement (10000 permutations) und Family-wise error rate correction for multiple comparisons. Color spectrum giving p-value indication (g).

The pattern of white matter damage significantly differed between bulbar- and limb-onset ALS (**figure 5**). Those with bulbar-onset demonstrated more widespread intensity change, extending into frontal and parietal regions, whereas changes in those with limb onset were most pronounced in the CST.

## Discussion

This study employed a novel and sensitive method for detecting white matter pathology in ALS, based on T1 images that could be acquired from a routine clinical scanner, in theory as part of the patient's diagnostic work-up. The main results are:

- VBI captures the core ALS white matter change previously established in DTI studies.
- VBI changes correlate with global disability.
- VBI reveals a significantly different pattern of white matter involvement between bulbar- and limb-onset patients.

ALS related white matter pathology, demonstrated by VBI increases, was observed in motor and extra-motor areas. In particular in the CST, corpus callosum, frontal and occipital corona radiata, cerebellum and periaqueductal regions. Such widespread white matter involvement is entirely in keeping with the acceptance of ALS as a multi-system disorder, and the large number of published studies using DTI [20]. The involvement of the posterior limb of the internal capsule that we observed has been previously highlighted in relation to prognosis [21]. The changes in frontal areas are in line with bilateral frontal atrophy in VBM [8], [10], [12], [13], an frontally increased diffusivity [22], [23] and *post mortem* studies [24]. Also VBI confirms that the corpus callosum, which connects orbitofrontal and frontal cortices, is consistently involved in ALS pathology [25]. This included the genu as well as the body, as observed in relation to cognitive impairments in a study cerebral myelin integrity in ALS [26], [27]. Together with microstructural changes in the hippocampal formations, cingulum, and frontal white matter, these wider VBI changes may reflect frontotemporal cognitive impairment, which is detectable in up to 50% of patients with ALS even though frank dementia (excluded in our study) is much rarer [28].

VBI changes were also sensitive to disease severity (ALSFRS-R), which may offer potential as an objective monitoring marker in therapeutic trials, or in facilitating prognostic stratification. Clinical correlations in ALS neuroimaging studies have been surprisingly variable, so this requires replication in another cohort. Future studies should also consider any potential relationship to the precise mix of clinical upper and lower motor neuron signs, which were not captured in this study. Epidemiological studies have identified prognostic subtypes of ALS [29]. Different structural and functional patterns of cerebral involvement between bulbar- and limb-onset ALS were previously demonstrated with advanced imaging methods [20], [30]. However, the more widespread changes we observed in the bulbar-onset might reflect higher burden of cognitive impairment in this subgroup that we did not capture in this study. However the question on how and why several ALS subtypes differ cannot be fully answered here. Although our patients did not suffer from frank dementia, neuropsychological dysfunction is an important additional factor, which we cannot rule out in the current study, because extensive neuropsychological testing was not undertaken.

Although the observed intensity changes in T1, which we found by using VBI are an amalgamation of different effects such as a shortening in T1 relaxation time, we hypothesize that the observed changes in T1 white matter intensity might be attributable to an increase in proton density. Quantitative analysis of proton density images in ALS supports this view [31]. Such changes might relate to increased microglial activity, intra-axonal neurofilament accumulation and gliosis occurring as part of neuroinflammatory processes accompanying motor neuron degeneration [32]–[35].

Traditional VBM has evolved to try and circumvent bias by assigning tissue probabilities to single voxels. This may be appropriate for conditions where regional grey matter atrophy is a dominant and defining pathological feature. VBI implements coarse bias correction through applying techniques such as bias fields and global scaling, hence retaining valuable intensity information in deep white matter areas that are far more pertinent to ALS pathology. Our VBI analysis currently lacks the quantification potential of DTI. Nevertheless VBI has the advantage of being a potentially much easier sequence to standardize and harmonize across multiple centers, which is a major requirement for the translation of candidate MRI-based biomarkers into future therapeutic trials [36]. Longitudinal studies using VBI have to be done, and advances in *post mortem* MRI [37] potential for the histological validation of VBI changes. The study of VBI in relation to more detailed assessment of regional involvement, as well as cognitive change, will also be important future studies. The challenge of individual rather than group-based analysis remains the major obstacle to clinical translation, and the prospective testing of assessor-blinded scans against a larger control database is one immediate way forward. These challenges notwithstanding, we suggest that VBI has significant biomarker potential in ALS.
